# The potential of cold-shock promoters for the expression of recombinant proteins in microbes and mammalian cells

**DOI:** 10.1186/s43141-022-00455-9

**Published:** 2022-12-29

**Authors:** Yaneth Bartolo-Aguilar, Cipriano Chávez-Cabrera, Luis Bernardo Flores-Cotera, Jesús Agustín Badillo-Corona, Carmen Oliver-Salvador, Rodolfo Marsch

**Affiliations:** 1 Department of Biotechnology and Bioengineering, Cinvestav-IPN, Av. Instituto Politécnico Nacional 2508, Col. San Pedro Zacatenco, 07360 Mexico City, Mexico; 2 Instituto Politécnico Nacional-Unidad Profesional Interdisciplinaria de Biotecnología, Av. Acueducto s/n, Colonia Barrio La Laguna Ticomán, 07340 Mexico City, Mexico; 3 Colegio de Estudios Científicos y Tecnológicos del Estado de Michoacán, CECyTE Michoacán, Héroes de la Revolución S/N, Col. Centro, 61880 Churumuco de Morelos, Michoacán Mexico

**Keywords:** Cold-inducible promoter, Aggregation-prone recombinant protein, Active enzyme, Unstable gene product, Protein stability, Protein quality

## Abstract

**Background:**

Low-temperature expression of recombinant proteins may be advantageous to support their proper folding and preserve bioactivity. The generation of expression vectors regulated under cold conditions can improve the expression of some target proteins that are difficult to express in different expression systems.

**Main body of the abstract:**

The *cspA* encodes the major cold-shock protein from *Escherichia coli* (CspA). The promoter of *cspA* has been widely used to develop cold shock-inducible expression platforms in *E. coli*. Moreover, it is often necessary to employ expression systems other than bacteria, particularly when recombinant proteins require complex post-translational modifications. Currently, there are no commercial platforms available for expressing target genes by cold shock in eukaryotic cells. Consequently, genetic elements that respond to cold shock offer the possibility of developing novel cold-inducible expression platforms, particularly suitable for yeasts, and mammalian cells.

**Conclusions:**

This review covers the importance of the cellular response to low temperatures and the prospective use of cold-sensitive promoters to direct the expression of recombinant proteins. This concept may contribute to renewing interest in applying white technologies to produce recombinant proteins that are difficult to express.

**Graphical Abstract:**

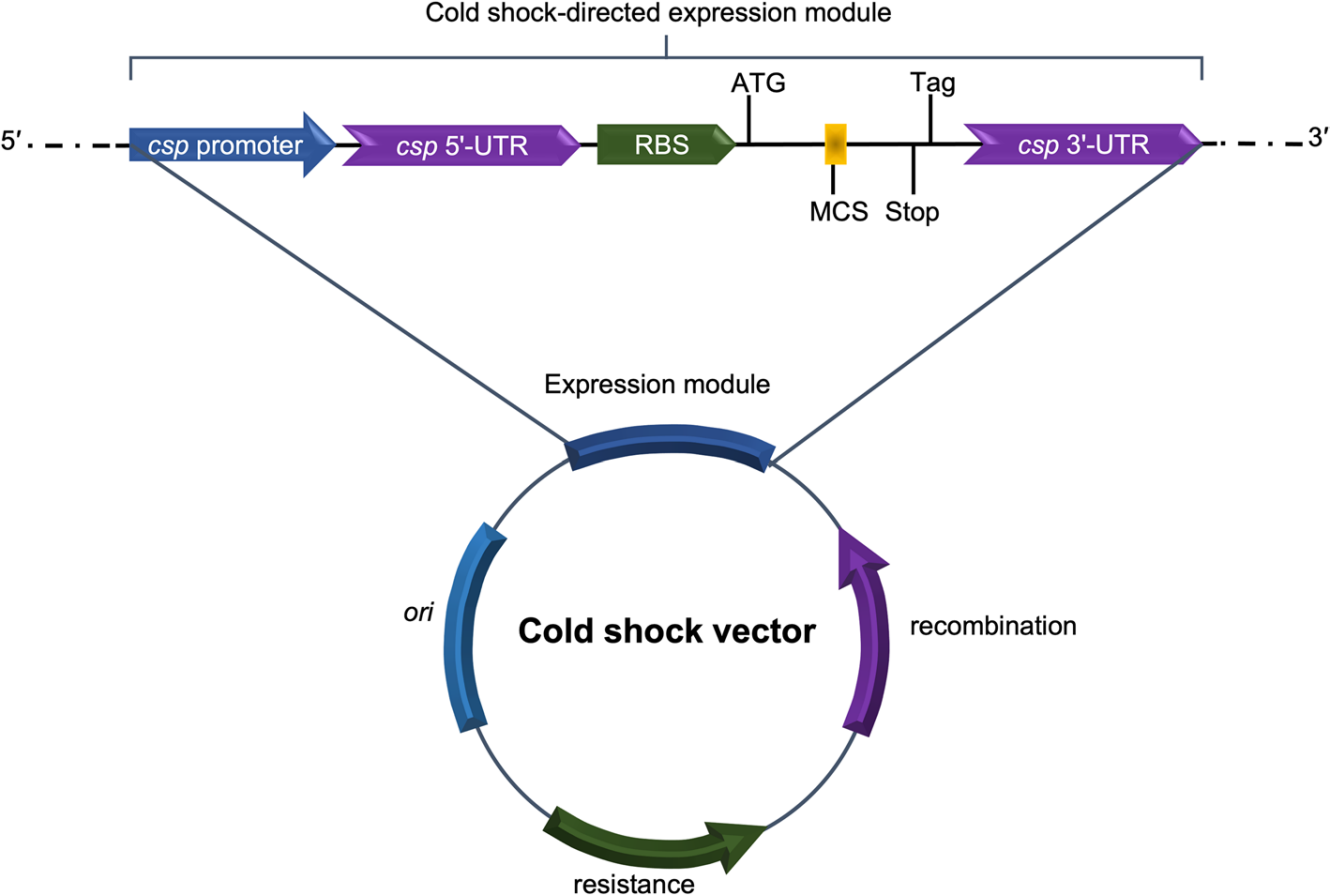

## Background

With the development of recombinant DNA technology in the 1970s, the expression and production of recombinant proteins in various host organisms became feasible, easier, and more cost-effective than proteins derived from natural sources. *Escherichia coli* has been a widely used bacterium for the expression of recombinant proteins, mainly due to its multiple advantages such as fast growth, high yield, low production costs, easy genetic manipulation, and availability of multiple molecular tools [1–3]. Other hosts are also employed for technical reasons or to improve the quality of the expressed proteins. For example, prokaryotic organisms do not perform complex post-translational modifications compared to eukaryotic organisms. In addition, some toxic components of bacteria may be of concern, mainly when a given protein is intended for therapeutic use. In contrast, some yeasts are known to have a GRAS (generally recognized as safe) status according to the Food and Drug Administration (FDA), making them well-suited for recombinant protein production [1, 4–6]. To make human-like proteins, yeast cells have been engineered to attach glycosylated side chains into recombinant proteins [7]. Moreover, mammalian cells are the preferred hosts for expressing high-quality eukaryotic proteins, that is, proteins similar or identical to those of the original host [3, 7].

Recombinant proteins are sometimes difficult to produce in their functional form using established expression systems. Thus, it is often necessary to optimize their production in the host system, e.g., to minimize the formation of inclusion bodies in *E. coli.* Several other strategies have been developed. Besides, protein expression at low temperatures has been reported to significantly influence product quality [1, 2, 8]. Nonetheless, the use of low expression temperatures decreases both, cell growth and target protein yield. These drawbacks can be circumvented with the use of cold-inducible promoters to induce recombinant protein production at low temperatures [1, 9, 10].

The heat shock response has been well documented in prokaryotic and eukaryotic organisms. Also, the cold-shock response has been studied in different organisms [11, 12]. Both prokaryotic and eukaryotic cells develop an adaptive cold shock response when faced with a sudden drop in temperature. This response often results in a loss of protein synthesis capacity, except for the transient overexpression of a small group of proteins called CSPs (cold-shock proteins). Certain CSPs enable an accurate and enhanced translation of low-temperature-specific messenger RNAs (mRNAs) [11, 13, 14]. Some studies have shown that the response varies, mostly depending on temperature and period at low temperature [12, 15]. In *E. coli*, the cold shock response enables cell survival, and ultimately, allows cells to resume growth at unfavorable low temperatures by modulating DNA replication, transcription, translation, stabilization of RNA, and ribosome assembly [16]. In *E. coli*, the induction mechanism and expression control of CspA have been extensively studied [13, 17, 18]. Contrasting, the molecular response to cold shock has been less studied in eukaryotes. Nonetheless, several studies have shown that, as in prokaryotes, CSPs induction in eukaryotes is essential for cell survival, adaptation and growth at low temperatures [13, 15, 19]. Unlike their bacterial counterparts, yeast cells show a more moderate cold-shock response as the temperature approaches 10 °C. Some cold-inducible genes have been identified in the budding yeast *Saccharomyces cerevisiae* [11, 20]. Yeasts respond to challenging low temperatures by tuning the expression of approximately 25% of their total genes, i.e., largely by upregulating genes involved in the synthesis of ribosomal RNA (rRNA), ribosomal proteins, and various stress response proteins. Furthermore, yeasts strongly inhibit their growth as the temperature approaches the freezing point [11, 21, 22]. Several studies have shown that yeasts and mammalian cells when exposed to sub-physiological temperatures, that is, below the optimum growth temperature, develop an adaptive response to regulate, in a hierarchical and coordinated manner, the cellular processes that affect cell growth such as transcription, translation, and metabolism [15, 23, 24]. This work aims to review the importance of the cellular response in bacteria, yeasts, and mammalian cells when confronted with low temperatures to document the potential of the use of cold-sensitive promoters for the development of genetic platforms or expression vectors, regulable at low temperatures, to produce recombinant proteins.

## Expression systems of recombinant proteins

Certain considerations are crucial to achieving high yields of recombinant proteins. Some key factors include using a suitable expression system, optimal culture conditions, and the availability of genetic tools expedient to the individual recombinant protein expressed [7, 25]. Commercial platforms, either with constitutive or inducible gene expression promoters, ease the expression of recombinant proteins because of the different genetic tools they provide for their utilization.

In the pharmaceutical industry, the bacterium *E. coli* and the mammalian cell lines HEK (human embryonic kidney), and CHO (Chinese hamster ovary) are commonly used for the expression of recombinant proteins. The yeasts *S. cerevisiae* and *Pichia pastoris* are being increasingly used, while transgenic plant cells are barely used [3, 26–30]. The choice of expression system depends largely on the sought characteristics in the expressed protein, the genetic engineering tools available, and economic factors. Protein quality is of paramount importance when it comes to proteins intended for pharmaceutical use, or whenever a biologically active protein is required [7].

### Bacterial expression systems

The *E. coli* expression system has some convenient features, such as high growth rate, high achievable cell density in culture, grows on simple culture media, and high level of recombinant protein expression. In addition, different expression platforms and engineered strains are available for special purposes, for example, for fast and easy genetic transformation. *E. coli* has doubling time of 20 min in Luria-Bertani broth and can reach maximum cell density of up to 200 g (DCW)/L, or near to 1 × 10^13^ viable bacteria/mL [6, 25, 31, 32]. Unlike *E. coli*, *Bacillus subtilis* has a GRAS status and is able to express extracellular proteins, consequently it is the most studied Gram-positive bacterium. Other advantages of this expression system are its well-characterized genetics, its short fermentation time, and ability to grow in low-cost culture media, making it ideal for industrial and pharmaceutical applications. However, the often-low efficiency of genetic transformation and the lack of molecular biology tools have limited the application of the protein expression systems based on *Bacillus* [33, 34]. Although bacterial systems offer many advantages, not all proteins can be suitably or readily expressed with them. Overall, they suffer from plasmid instability, and are markedly deficient when complex post-translational modifications are needed. Besides, some proteins can form inclusion bodies, which may require additional processing steps for refolding. As a result, this may substantially increase costs or undesirably affect yield. Furthermore, the possible accumulation of endotoxins, pyrogenic to humans or animals, is another disturbing drawback when therapeutic proteins are produced in *E. coli* [1, 6, 29]. Thereby, a continued search is required to find other suitable expression systems to increase either the quality, quantity, or stability of recombinant proteins.

### Yeast expression systems

Yeasts are well-known hosts for the expression of recombinant proteins and glycoproteins for therapeutic use, mainly because they are regarded as inexpensive and easy-to-use systems. In addition, yeasts offer significant advantages over their bacterial counterparts, in that they can perform some complex post-translational modifications [35]. Unfortunately, yeasts as a rule bind heterogeneous high-mannose glycan side chains on the recombinant glycoprotein, which may cause immunogenic reactions in humans, e.g., the typical α-1,3-linked mannose modifications of *S. cerevisiae*. For this reason, glycosylation pathways have been engineered in different yeast expression systems to yield human-like glycosylation patterns and thus avert the side effects of unwanted post-translational modifications in therapeutic proteins [36–38]. Although *S. cerevisiae* typically produces hyperglycosylated recombinant proteins, it is currently the most widely used host to produce yeasts-derived therapeutics [36, 38]. Other yeast expression systems, such as *P. pastoris* produce recombinant proteins with a moderate degree of glycosylation. *Pichia pastoris* is obligate aerobic, and a Crabtree-negative yeast, which can be grown to high cell density and, consequently high protein yield [7, 35]. There are numerous commercial platforms available with strong and inducible expression promoters for *P. pastoris*. As a result, this species is also widely used in the pharmaceutical industry [35, 39, 40]. Other yeasts used for recombinant protein production include *Hansenula polymorpha*, *Yarrowia lipolytica*, *Arxula adeninivorans*, *Kluyveromyces lactis*, and *Schizosaccharomyces pombe* [7, 36, 41, 42].

### Mammalian cell expression systems

Most FDA-approved human therapeutic recombinant proteins have been produced in mammalian cell lines. HEK293 and CHOK1 cells are the two most widely used cell lines for recombinant protein production. Multiple laboratories worldwide have fitted cell lines to grow in suspension at high cell densities. Productivity has been dramatically increased by using viral elements in these two cell lines [7, 43, 44]. A decisive advantage of human cell lines is that the resultant recombinant protein carries post-translational modifications more consistent with endogenous human proteins [45]. Mammalian cell lines have also been developed to produce humanized glycosylation patterns into some recombinant therapeutic products [7].

## Regulation of gene expression

**Fig. 1:**
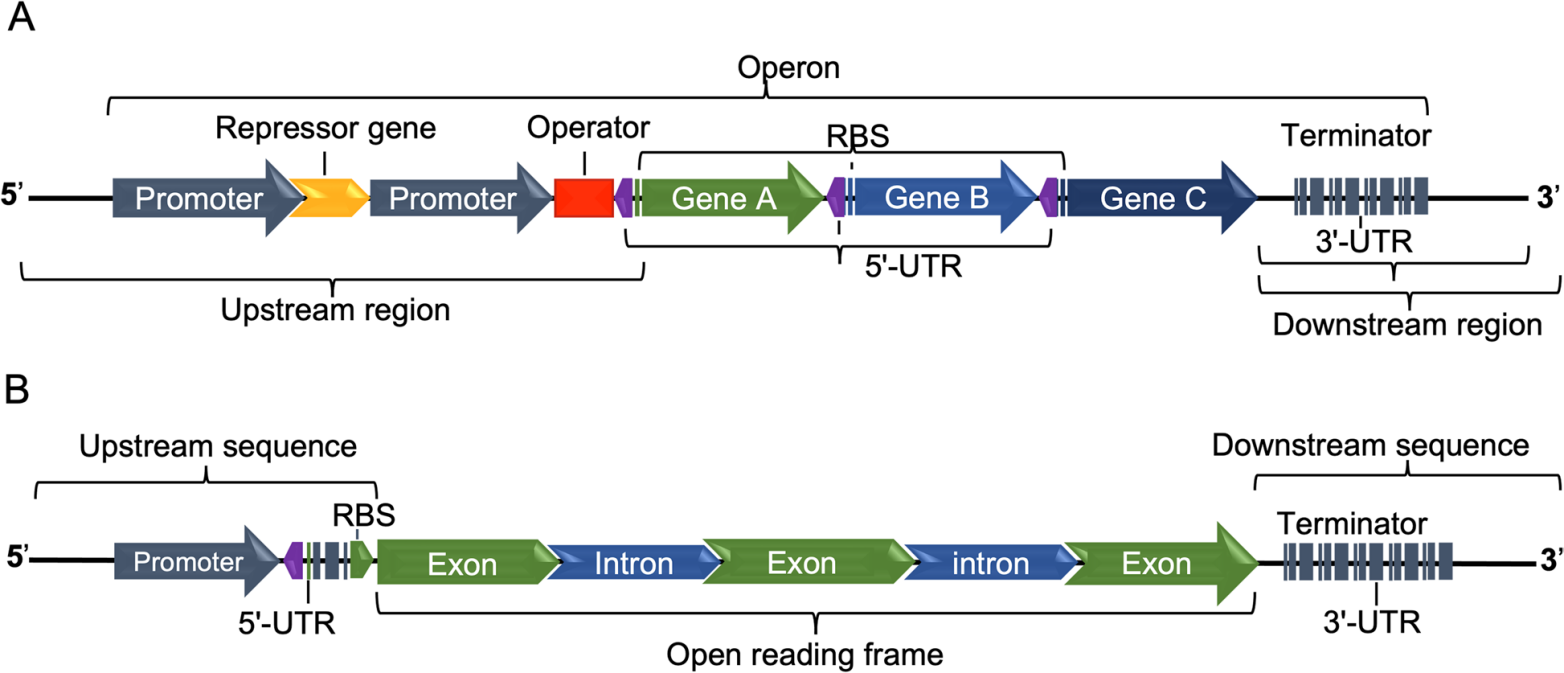
Structure of prokaryotic and eukaryotic genes. **A** and **B** show the elements that comprise the canonical genes. **A** In prokaryotic genes, the regulatory gene elements for transcription are sited upstream, downstream, and on regulatory sequences such as RBS and intergenic UTRs. **B** In eukaryotic genes, the regulatory gene elements for transcription are sited upstream, downstream, and intronic regions. 5′-UTR: 5′-untranslated region, 3′-UTR: 3′-untranslated region, RBS: ribosome binding site

Genetic regulation is turning genes on and off at appropriate times to let cells adaptively respond to their environment. This regulation includes the recruitment and binding of regulatory proteins onto the DNA regulatory elements of genes. Regulatory proteins, named transcription factors, can facilitate the recruitment of the RNA polymerase to the transcription start site (TSS) [46, 47]. A promoter is a regulatory region of DNA sited upstream of prokaryotic and eukaryotic genes, which contains the RNA polymerase binding site, the TSS, the transcription factor binding sites (TFBs) and transcription enhancer elements (TEEs). In *E. coli*, promoters typically consist of three regions named the -35 and the -10 boxes and a spacer region of 17 nucleotides separating said boxes. The promoter has a consensus sequence TTGACA-N17–TATAAT, where N17 represents the spacer region. Some promoters contain a fourth region, the UP element (upstream element), located upstream of the -35 box. This UP element is an AT-rich sequence that allows binding to the C-terminal domain of the RNA polymerase α subunit to increase promoter strength [47, 48]. In prokaryotes, proteins needed for a biosynthetic pathway are encoded together in specific DNA segments called operons (Fig. 1A). In *E. coli*, the lactose operon (*lac* operon) regulates the expression of genes (named polycistronic) that encode the enzymes necessary for lactose catabolism. The *lac* operon consists of structural genes (*lacZ*, *lacY*, and *lacA*) and a promoter sequence that includes an operator sequence [46]. Both, the LacI repressor and catabolite activator protein (CAP) regulate the operon in a lactose and glucose level-dependent manner. Transcription of structural genes is prevented by the binding of the LacI repressor protein to the operator sequence, so that, the RNA polymerase binding to the *lac* promoter is prevented, and transcription cannot occur. The presence of lactose through its allolactose isomer causes the LacI repressor to disassociate from the operator sequence (by binding allolactose/LacI repressor); this promotes a greater RNA polymerase affinity to the promoter and gene transcription [49]. The sugar chemical analog isopropyl β-D-1-thiogalactopyranoside (IPTG) is also often used to induce the expression of recombinant proteins, under the control of the *lac* promoter, in transformed cells [50]. Knowledge on the *lac* promoter function has made feasible to engineer the promoter to generate novel promoters with greater expression strength. For example, the mutant *lacUV5* promoter, differed by two nucleotides from the -10-consensus box of the *lac* promoter, but showed 2.5-fold greater strength. Based on the *lacUV5* and *trp* (tryptophan) promoters, an artificial promoter consisting of the -35 consensus sequence of the *trp* promoter and the -10-consensus sequence of the *lac* promoter was constructed and named *tac* promoter [51]. The *trc* promoter differs from *tac* promoter only by one nucleotide. The *tac* and *trc* promoters allowed polypeptide accumulation between 15 and 30% of the total cellular protein [52]. The commercially developed T7 expression platforms hold the T7 promoter, which binds T7 RNA polymerase. *Escherichia coli* BL21 (DE3) produces recombinant T7 RNA polymerase, and its chromosome carries one copy of the bacteriophage T7 gene 1 under the regulation of the IPTG-induced *lacUV5* promoter [53]. Moreover, the engineered *lac* promoter rendered a series of PAR promoters that showed lower gene expression strength, which is key to decrease the expression of toxic proteins and to improve solubility of aggregation-prone proteins [54]. Table 1 shows some of the most used promoters in commercial and research platforms for the overexpression of recombinant proteins in bacteria, yeasts, and mammalian cells.

**Table 1:** Some commercially available platforms for *Escherichia coli*, yeasts, and mammalian cells

Strain	Commercial platform	Promoter	Reference
*Escherichia coli*			
BL21 (DE3)	pET (Novagen, USA)	T7	[55]
BL21 (DE3)	pRSET (Invitrogen, USA)	T7	[56]
ER2566	pBAD24 (Invitrogen, USA)	*araBAD*	[57]
M15	pQE (Qiagen, USA)	T5/*lac*	[58]
BL21 (DE3)	pTrcHis (Invitrogen, USA)	Trc/*lac*	[59]
BL21 (DE3)	pTAC-MAT-Tag-2 (Sigma-Aldrich, USA)	*tac*	[60]
*Pichia pastoris*			
X-33	pPICZB (Invitrogen, USA)	*AOX1*	[5]
KM71H and X-33	pPICZαA (Invitrogen, USA)	*AOX1*	[61]
SMD1168	pGAPZαA (Invitrogen, USA)	*GAP*	[62]
*Kluyveromyces lactis*			
GG799	pKLAC1 (New England Biolabs, USA)	*LAC4*	[63]
*Saccharomyces cerevisiae*			
BY4741	pYES2 (Invitrogen, USA)	*GAL1*	[64]
INVS1	pYES2/NT A (Invitrogen, USA)	*GAL1*	[65]
Mammalian line cells			
EXPI293F	pcDNA3.3-TOPO (Invitrogen, USA)	CMV	[66]
HEK293T	pLenti6.3/TO/V5-DEST (Invitrogen, USA)	CMV/TO	[67]
C2C12	pGene/V5-His (Invitrogen, USA)	*GAL4*-E1b	[68]

*Abbreviations*: T7, T7 RNA polymerase; *araBAD,* arabinose operon; *T5/lac*, *lac* operator sequence just downstream of the T5 promoter; *trc*/*lac*,* trc* promoter is a hybrid between the *trp* (tryptophan) and *lac*UV5 promoters containing de *lac* operator; *tac*, the *tac* promoter is a synthetic DNA promoter, produced from the combination of promoters from the *trp* and *lac* operons; *AOX1*, alcohol oxidase 1 gene; *GAP*, glyceraldehyde-3-phosphate dehydrogenase gene; *LAC4*, β-galactosidase gene; *GAL1*, galactokinase gene; CMV, human cytomegalovirus; GAL4-E1b, the adenoviral E1B minimal core promoter fused to DNA-binding sites for the yeast GAL4 DNA-binding protein

Each eukaryotic gene, in contrast to those of prokaryotes, as a rule has its own promoter at the 5′ end and a transcription terminator at the 3′ end, i.e., eukaryotic genes are monocistronic. The complexity of eukaryotic cells depends on the relative amount of noncoding DNA in their genomes. Noncoding regions regulate specific gene expression by combining numerous mechanisms. Overall, each eukaryotic gene is regulated mainly by means of the upstream and downstream regions, and introns, as shown in Fig. 1B. These regulatory regions are affected by various noncoding transcripts generated during the transcription of complex genomes [69, 70]. The binding of the transcription factor IID (TFIID) to the core promoter, which comprises ~ 80 bp around the TSS of eukaryotic genes, allows the recruitment of the transcription initiation complex, the subsequent binding of RNA polymerase II and transcription initiation. Mammalian core promoters can be lumped into conserved TATA-box enriched promoters and variable CpG-rich promoters containing single or multiple TSS, respectively. Some of the genetic elements involved in regulation by these complex promoters are enhancers, TFIID recognition elements, initiator elements, insulators, activators, silencers, repressors and other elements. Many mammalian genes have alternative promoters that markedly affect gene expression and may produce different specifically expressed mRNA isoforms. This mechanism interferes with the cell-specific expression and the development-specific expression of many genes [69, 71].

The 5′ untranslated region (UTR) is sited at the 5′ end of all protein-coding genes. 5′-UTR mRNAs play regulatory roles in the control of translation initiation and contain several regulatory elements, such as secondary structure of mRNA, which affects the ribosome binding site (RBS) accessibility [69, 72]. Prokaryotic mRNA 5′-UTRs are much shorter than those of their eukaryotic counterparts. Throughout the eukaryotic evolution, the length of the 5′-UTR increased, and then regions most likely adopted more stable secondary structures. The average length of 5′-UTRs is around 53 nucleotides in the budding yeast and 218 nucleotides in humans. However, the length of the 5′-UTR can vary from a few to thousands of nucleotides in higher eukaryotes [73]. Other regulatory elements affecting gene expression include the RBS, the Shine-Dalgarno (SD) sequence in prokaryotes, and the Kozak sequence in eukaryotes. The SD sequence has a consensus sequence 5′-AGG AGG-3′ located 3–9 bp from the translation start codon. It serves to correctly dock the ribosome on the mRNA. In eukaryotes, the 5′-UTR nucleotide composition varies depending on the gene type and species. The Kozak sequence, sited 6–9 nucleotides upstream of the translation start codon, has a consensus sequence 5′-WAMAMAA-3′ in yeast, and 5′-GCCGCCRMC-3′ in humans [74]. The eukaryotic 5′-UTR also contains other regulatory elements, such as the 5′-cap structure, upstream open reading frames (uORFs), upstream AUGs (uAUGs), secondary structure (including internal ribosomal entry sites, IRES), TFBs, and intronic regions (reviewed by [42, 69, 75]). The 3′-UTRs are located at the 3′ end of genes and works together with the 5′-UTRs to regulate translation initiation and decay of their own mRNAs. However, the 3′-UTR may be prone to attack by ribonucleases, especially the long 3′-UTRs [76, 77]. The bacterial mRNA 3′-UTR is a rich source of functional small RNA (sRNA) with predicted roles in many physiological circuits [78]. In addition, 3′-UTRs act as sRNA targets to influence their own gene expression by positively or negatively modulating mRNA stability. In eukaryotes, mRNA 3′-UTRs regulate gene transcription by modulating mRNA decay, translation, or localization [77]. The 3′-UTR contains binding sites for numerous regulatory proteins and microRNAs which interact with mRNA targets post-transcriptionally to decrease gene expression, either by inhibiting translation or directly causing mRNA degradation [69, 74]. Moreover, the 3′-UTR contains the A-rich positioning element that directs the addition of several hundred adenine residues called the poly(A) tail to the end of the mRNA transcript. The six-base consensus sequence 5′-AAWAAA-3′ is found in yeast and 5′-AAT AAA-3′ in humans. This polyadenylation allows mRNA binding to a class of regulatory factors called poly(A)-binding proteins, influencing mRNA export, stability, and translation. Other regulatory elements that contain a 3′-UTR are UA-rich efficiency elements and multiple U-rich sites [74].

## The cold-shock response

**Fig. 2:**
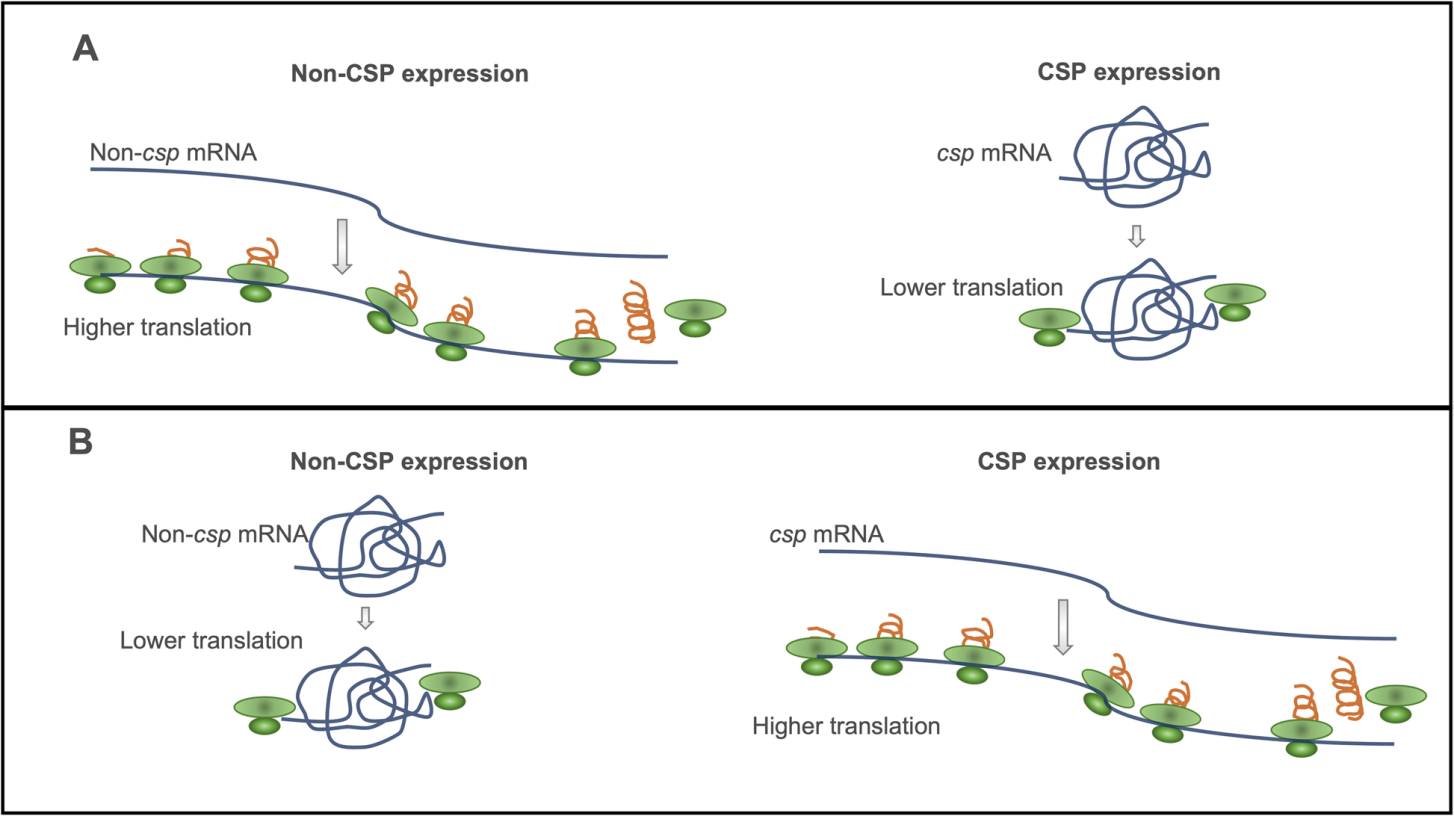
Effect of sub-physiological temperatures on CSP translation. **A** Protein translation at physiological temperature where the concentration of non-CSPs increases while the CSPs remain at low levels. **B** The cellular response under cold shock involves modulation of protein expression where the concentration of CSPs increases while the non-CSPs diminishes. Reduced expression of non-CSPs is linked to repression of non-*csp* mRNA translation, leading to slower growth and cell cycle arrest. CSP overexpression is linked to higher *csp*-mRNA translation due to a secondary structure change that facilitates ribosome docking. CSP overexpression allows cell growth to continue under cold stress. CSP: cold-shock protein

Environmental changes, such as temperature, are common challenges that cells repeatedly face. In particular, the response to cold stress has not been fully characterized [79, 80]. However, it is generally accepted that prokaryotic and eukaryotic cells counteract the effects of low temperatures by decreasing transcription and translation. At the same time, some specific genes, the so-called cold-shock proteins (CSPs), are upregulated (Fig. 2), promoting the continuity of the cell cycle [17, 79, 80]. Subsequently, cells modulate membrane fluidity by adjusting its composition, e.g., by increasing the content of polyunsaturated fatty acids in the membrane phospholipids. These and other molecular changes allow cellular adaptation to a cold shock. The production of cold shock-inducible proteins is fundamental for cellular adaptation at low temperatures, but it may be also essential under normal growth conditions [13, 17, 81–84]. Bacterial CSPs are small proteins having a single cold-shock domain (CSD), while their eukaryotic homologs may possess one or more CSDs and are of variable length. All these domains bind to single-stranded nucleic acids. Some biological functions of proteins containing CSDs comprise DNA repair and transcriptional regulation, control of mRNA splicing, stability, translation, and sequestration. Bacterial CSPs and eukaryotic CSDs are similar in length and share conserved sequences [81].

### The cold-shock response in bacteria

Cold-shock proteins are a family of small nucleic acid-binding proteins that range from 67 to 75 amino acids in length [85]. CSPs with highly conserved sequences (> 45% identity) have been identified in many Gram-positive and Gram-negative bacteria, including psychrophilic, mesophilic, thermophilic, and even hyperthermophilic bacteria. More specifically, they are found in *B. subtilis*, *Bacillus cereus*, *E. coli*, *Clostridium botulinum, Listeria monocytogenes*, *Yersinia enterocolitica*, *Yersinia pseudotuberculosis*, and *Thermus thermophilus* [86–88]. CSPs are strongly induced to mitigate the deleterious effects that low temperatures could cause [87]. When *E. coli* cells face a drastic drop from 37 to 15 °C, there is an immediate CSP synthesis response. During the acclimatization stage, the concentration of CSPs increases while the concentration of non-CSP proteins diminishes. Simultaneously, the cell growth rate decreases, but towards the end of the acclimatization stage, cell growth resumes while the concentration of CSPs decrease in parallel with a rise of non-CSP proteins (Fig. 2). Activation of CSP synthesis allows cell growth to continue, although at a slower pace [20, 87]. It should be noted that not all CSPs are induced only by cold, which suggests that certain members of the CSP family can participate in other cellular processes [86, 89]. CspA was the first and major CSP described in *E. coli* [87, 90]. CspA has been studied regarding its structure, function, transcriptional regulation, translation, and mRNA stability. The *E. coli* CspA family consists of nine homologous proteins, CspA to CspI, among them, CspA, CspB, CspE, CspG, and CspI are inducible by cold shock and play a major role in low-temperature adaptation [87, 91]. Several essential functions have been attributed to CSPs during adaptation to cold and general stress, i.e., transcriptional activators, RNA chaperones, protection against cold shock, and freezing [87, 92]. The role of CSPs as RNA chaperones was revealed due to the highly conserved RNA-binding motifs found in the *E. coli* CspA [14, 87]. Similarly, Graumann et al. [86] showed that the CSP family from *B. subtilis*, i.e., CspB, CspC, and CspD, cooperatively and interactively bind to RNA, suggesting that CSPs function as RNA chaperones that enable translation initiation at low and optimum temperatures. This family of CSPs is essential for the cell growth of *B. subtilis* and for efficient protein synthesis at optimal temperature, besides, an increased CSPs synthesis is crucial for cold acclimatization. Hunger et al. [93] reported that cold-induced putative DEAD-box RNA helicases CshA and CshB work together with CSPs to facilitate ribosomal translation initiation in *B. subtilis*. In addition, the *E. coli* CspA acts as a cold-shock transcriptional activator of the genes encoding DNA gyrase GyrA and nucleoid protein H-NS. This shows that CspA favors RNA polymerase binding to the promoter region of target DNA [92, 94, 95].

The 5′-UTR of mRNAs commonly promotes the formation of stable secondary structures upon cold stress, which may prevent access of the SD sequence to ribosomes, and therefore, the initiation of translation. This secondary structure can also be a target for RNaseIII. Nevertheless, it was suggested that the *Bacillus caldolyticus* CspB might induce its own translation under cold shock by destabilizing the 5′-UTR mRNA secondary structure, thereupon increasing translation efficiency and inhibiting mRNA decay [17]. Likewise, it was suggested that the *B. caldolyticus* CspB might induce other CSPs under cold shock. Mega et al. [88] used DNA microarrays to evaluate the expression of *ttcsp2* mRNA from the thermophilic bacterium *Thermus thermophilus*. Besides, they predicted the structure adopted by the 5′-UTR of *ttcsp2* mRNA as the temperature decreased. The *ttcsp2* mRNA of *T. thermophilus* acts as a thermosensor that rapidly changes its secondary structure when the temperature drops. Moreover, Giuliodori et al. [96] showed that the *cspA* mRNA of *E. coli* undergoes analogous changes in its secondary structure after a drop-in temperature and may adopt different functional secondary structures. This allows more efficient translation of the *cspA* mRNA than that occurs at 37 °C. Ivancic et al. [97] studied the physiological response of *E. coli* when challenged by slow periodic temperature fluctuations between 37°C and 8°C. Several cold-stress response proteins were significantly up- or downregulated with each temperature cycle, including cold-shock proteins CspA and CspB, as well as proteins involved in energy metabolism, transport proteins, and amino acid biosynthetic proteins. Thereby the *cspA* and *cspB* mRNA transcripts increased following each drop-in temperature and decreased dramatically after each increase in temperature.

The half-life of *E. coli cspA* mRNA was 12 s at 37 °C but increased to 20 min after a cold shock. These data suggested that the *cspA* mRNA became more stable at low temperatures [87]. Palonen et al. [98] presented a possible regulatory mechanism of the CspA expression in *E. coli*. However, the regulation of CSP expression in response to cold shock has not been fully elucidated. Table 2 shows the major genes upregulated by cold shock in bacteria, i.e., those induced when cells are transferred from an optimal growth temperature to a lower temperature.

**Table 2:** Major genes upregulated by cold shock in bacteria

Gene	Source	Reference
*cspA*	*Escherichia coli*	[90]
*cspB*	*Escherichia coli*	[99]
*cspE*	*Escherichia coli*	[100]
*cspG*	*Escherichia coli*	[101]
*cspI*	*Escherichia coli*	[102]
*deaD*	*Escherichia coli*	[103]
*gyrA*	*Escherichia coli*	[95]
*hns*	*Escherichia coli*	[104]
*nusA*	*Escherichia coli*	[14]
*recBCD*	*Escherichia coli*	[105]
*trmE*	*Pseudomonas syringae*	[106]
*hutU*	*Pseudomonas syringae*	[107]
*recBCD*	*Pseudomonas syringae*	[108]
*aat*	*Pseudomonas syringae*	[109]
*rpoS*	*Pseudomonas putida*	[110]
*deaD*	*Thermococcus kodakarensis*	[111]
*deaD*	*Methanococcoides burtonii*	[112]
*cspB*	*Bacillus subtilis*	[113]
*cshB*	*Bacillus subtilis*	[93]
*cspA* and *cspB*	*Caulobacter crescentus*	[114]
*ttcsp2*	*Thermus thermophilus*	[88]

### The cold response in yeasts

*Saccharomyces cerevisiae* cells when confronted to low temperatures induce the expression of genes involved in cold stress cellular response such as transcription, translation, metabolism, protein folding, and signal transduction, all of which affect cell growth and developmental processes. However, the rationale behind all this and the regulatory mechanisms involved are not fully characterized in yeasts [11, 115, 116]. The response to cold, which occurs mainly between 0 and 18 °C, leads to changes in membrane fluidity by adjusting the lipid composition, to reduced enzyme activity, to the production of more stable DNA and RNA secondary structures, and to substantially reduced protein synthesis [12, 15, 117]. Genome-wide expression analysis using cDNA microarrays showed that about 25% of the genes of the *S. cerevisiae* genome are involved in the response to cold shock from 30 to 10 °C. This genetic response is time-dependent and controlled by the cAMP-protein kinase A pathway. According to the expression profiles, the upregulated genes can be categorized into three phases. During the early to middle phases at low temperature, yeast cells recruit transcriptional machinery to primarily upregulate the expression of ribosomal genes and a large set of other protein genes involved in RNA metabolism. The genes involved in RNA polymerase I and rRNA processing are upregulated in the early phase, whereas genes involved in cytosolic ribosomal proteins are upregulated in the middle phase. In contrast, expression of genes linked with the general stress response are upregulated in the late phase where RNA synthesis genes and transcriptional regulation genes are transcribed [15]. *NSR1,* a cold-inducible gene encoding a nucleolin-like protein related to rRNA processing and ribosomal biosynthesis, was upregulated in the early phase [15, 118].

Table 3 shows some *S. cerevisiae* genes that are upregulated by a cold shock. It is known that some genes showed, also respond to global stress, such as HSP12 and HSP26 in yeasts. The *S. cerevisiae* chaperonin CCT (chaperonin containing the T-complex polypeptide–1 [TCP1]) is a cold-shock protein essential for cell growth. CCT comprises two subunits, CCTα and CCTβ, both required for the assembly of tubulin and actin. Using Northern blot analysis, Somer et al. [119] found 3- to 4-fold increase in *CCTα* mRNA levels after a cold shock (4 °C for 6 h). The expression response of *CCTα* transcripts was time-dependent. Moreover, we showed that the *CCTα* promoter region of *S. cerevisiae* is operatively functional in a recombinant *P. pastoris* to regulate, by cold shock, the expression of the target *eng* mRNA [120]. Strikingly, the expression of *eng* mRNA regulated by the *CCTα* promoter was 4.5-fold higher upon a cold shock (4 °C for 6 h) as determined by qRT-PCR analysis. The time-dependent response to cold shock of *S. cerevisiae* was comparable to the response of the recombinant *P. pastoris* carrying the *CCTα* promoter*.* This suggests that these two yeasts may share a similar cold shock adaptive response.

**Table 3:** Some genes upregulated by cold stress in *S. cerevisiae* and mammalian cells

Gene	Description	Temperature downshift	Reference
Yeast: *Saccharomyces cerevisiae*			
*TIP1*	Temperature shock inducible protein	30 to 10 °C	[121]
*NSR1*	Protein involved in ribosomal biogenesis and cell growth	30 to 10 °C	[118]
*TIR1, TIR2*	Serine-rich stress proteins	30 to 10 °C	[122]
*BFR2*	Protein involved in secretion	30 to 10 °C	[123]
*TPS1, TPS2, HSP104*	Trehalose-synthesizing enzymes, 104 kDa heat shock protein	30 to 4 °C and 30 to 0 °C	[22]
*OLE1*	Stearoyl-CoA 9-desaturase	30 to 10 °C	[124]
*TPI1, ERG10, IMH1*	Triose-phosphate isomerase, acetoacetyl coenzyme A thiolase, protein implicated in protein transport, respectively	30 to 10 °C and 30 to – 20 °C	[125]
*GPD1*	Main enzyme for glycerol production	30 to 4 °C	[126]
*HSP12, HSP26*	26 kDa heat shock protein	30 to 10 °C	[15]
*GLC3, GAC1*	Glycogen biosynthesis	25 to 4 °C	[127]
*CCTα, CCTβ*	chaperonin containing the T-complex polypeptide–1 [TCP1] *α* and *β* subunits	30 to 4 °C	[119]
*HSF1, SSE2*	Heat shock transcription factor, HSP70 family member	30 to 4 °C	[128]
Mammalian cells			
*CIRP*	Cold-inducible RNA-binding protein	37 to 32 °C	[80]
*RBM3*	RNA-binding motif protein 3	37 to 32 °C	[129]
*HSP72, HSP90*	72 kDa heat shock protein, 90 kDa heat shock protein	37 to 4 °C and 37 to 15 °C	[130]
*WAF1*	A cyclin-dependent kinase inhibitor	37 to 4 °C	[131]

### The cold response in mammalian cells

The response to cold stress in mammals is relevant for survival, preserving tissues and organs, and treating brain damage in the medical field. Mammalian cell cultures performed at reduced temperatures (< 37 °C) are often referred as cold shock cultures [132]. The quality and yield of recombinant proteins can often be improved in cultured mammalian cells, in vitro, at mild hypothermia (28–34 °C), regardless of whether the proteins are destined for biopharmaceuticals or the biotechnological industry [82, 133]. Mild hypothermia habitually increases the expression level of therapeutic recombinant proteins and favors their correct folding. These effects have been ascribed to a drop in global protein synthesis and the activation of specific microRNAs that might remove gene-specific translational limitations at such conditions [80, 134, 135]. The microRNA 483 (miR-483) is among the most upregulated miRNAs under mild hypothermia, both in CHO and HeLa cells, and has an activating role in cell survival processes [136]. Several studies have reported high recombinant mRNA levels at temperatures below 37 °C in CHO cells, an often-used cell line to produce recombinant therapeutic proteins [133, 135, 137–139]. In these cells, post-translational events occurred with greater fidelity and accuracy at 32 °C compared to 37 °C, resulting in a higher yield of a model reporter protein [134]. Al-Fageeh and Smales [11] proposed a model that explains the coordinated cellular response of mammalian cells confronted to mild hypothermia.

Mammalian cells grow at moderately low temperatures (25–35 °C), but at lower temperatures (0–10 °C) cell growth arrests [11]. In mammalian cells, as in microbial cells, cold responsive proteins have also been identified. The two best characterized cold-inducible proteins are the cold-inducible RNA-binding protein (CIRP) and the RNA-binding motif protein 3 (RBM3). These two proteins share a high similarity and belong to the conserved family of glycine-rich RNA binding proteins [11, 23]. Both proteins can function as chaperones, like those of bacteria, preventing the formation of secondary RNA structures and easing protein translation. Therefore, both CIRP and RBM3 are regarded as modulators of transcription and translation, acting by different pathways during moderate hypothermic conditions or in other kinds of cellular stress [11, 13, 23, 82, 140]. Homologs of CIRP have been identified in mice, rats, and humans. The overexpression of CIRP at low temperatures delays the cell cycle and cell growth in mouse cells [11, 13]. CIRP provides neuroprotection via its intracellular activity, while its extracellular activity is detrimental because it boosts the inflammatory response [141]. Mouse RBM3 has a molecular weight of 18 kDa and shares 94% amino acid sequence identity with that of human RBM3 [11]. A crucial role of RBM3 is to protect neuronal cells by suppressing polyglutamine-induced cell death. Recently, RBM3 has attracted significant attention due to its critical protective role in hypothermia [141]. Table 3 shows some typical mammalian genes that are upregulated following a cold stress. Although the response to cold stress in mammalian cells has been less studied than in bacteria, yeasts, or plants, it is known from a few genomic, transcriptomic, and proteomic analyzes that the mammalian cellular response involves: (a) reduction in transcription and translation; (b) reduction of RNA degradation; (c) increased expression of cold-inducible genes; (d) the possible generation of alternative mRNAs; and (e) use preferential cap-independent translation of specific mRNAs [80, 82, 142–146]. Knowledge of specific molecules involved in the cellular response at subphysiological temperatures can be valuable to improve the expression of recombinant proteins. For example, Emmerling et al. [136] suggest that the co-expression of a target protein together with miR 483 improves the productivity at mild hypothermia.

## Cold shock promoters used for the expression of recombinant proteins

Given the trend toward the use of white technologies in industrial bioprocesses and the convenient characteristics of cold-sensitive promoters to produce recombinant proteins, the potential application of these promoters will be briefly mentioned in this section. To our knowledge, the promoter of the *cspA* gene of *E. coli* has been the only cold-induced promoter exploited commercially. However, the promoters of other cold-upregulated genes could also be suitable to overexpress recombinant proteins in bacterial and eukaryotic expression systems. In addition, cold-sensitive promoters, such as the *cspA* promoter, may offer significant advantages by stabilizing the mRNA of the target gene, favoring its translation and post-translational events, and improving protein solubility. Other inducible promoters under study that could contribute to establishing bioprocesses with clean technologies are those inducible by aromatic amino acids, light, and xylose [147]. Contrasting to this approach, today the most frequently used inducible promoters in industry and research are those induced by IPTG.

### Cold shock-responsive promoters for protein expression in prokaryotes

A major drawback of overexpressing recombinant proteins in *E. coli* is that they often do not readily fold into their native conformation. Instead, they can quickly form aggregates under conventional expression conditions, that is, when chemically or nutritionally inducible promoters drive the expression at physiological temperatures. Recombinant proteins readily accumulate into so-called inclusion bodies, for this reason, alternative protocols have been developed to recover these proteins. To obtain a given amount of soluble protein, a common approach involves the production of larger amounts of the protein expressed as inclusion bodies. Additional processes to recover the later protein may include in vitro refolding, strain engineering to increase the concentration of molecular chaperones, or cell cultures at relatively low temperatures. Low-temperature protein expression in *E. coli* prevents the degradation of proteolytically sensitive products and restricts the overall protein synthesis [25, 148], this may result in a higher yield of soluble protein versus aggregation-prone proteins or unstable products. However, cell growth may be notably affected under these conditions. Alternatively, bacterial expression systems with vectors directed by cold-inducible promoters may be grown at low temperatures for short periods only. This strategy often leads to overexpression of the target protein with the advantages cited above for cultures performed fully at low temperatures, i.e., with greater productivity of the target protein [25, 149].

The *cspA* cold shock promoter and its regulatory elements have been well suited to direct and optimize, in *E. coli,* the expression of recombinant proteins at low temperatures [150–152]. Vasina and Baneyx [152] assessed the usefulness of the *cspA* promoter to drive the expression of *lacZ*. The *cspA* promoter (586 bp) consisted of the *cspA* promoter region followed by its authentic RBS and the initial 24 nucleotides of the *cspA* open reading frame. The synthesis of β-galactosidase was efficiently repressed at 37 °C, but it was rapidly induced at temperatures in the range of 30 to 15 °C, leading to a 3- to 5-fold increase in specific activity relative to the control cultures. Next, Vasina and Baneyx [153] compared the efficacy of the IPTG-inducible *tac* promoter with that of the *cspA* promoter for the expression of the aggregation-prone recombinant protein preS2-S′-β-galactosidase. The two promoters yielded similar expression of active form of the protein at 25 °C, indicating that the *cspA* promoter could efficiently supersede the synthetic *tac* system. Moreover, while the *tac* promoter was inefficient at 10 °C, the *cspA* promoter prompted a rapid synthesis of the active β-galactosidase homotetramer. These data reinforce that the *cspA* promoter can be particularly valuable for the expression of labile or highly aggregation-prone recombinant proteins at 10 °C. Nonetheless, a drawback of the *cspA* promoter was that 1–2 h after a drop-in temperature the CspA protein synthesis was repressed. Despite this, Vasina and coworkers [154] have shown that this repression can be prevented by employing host cells carrying a mutation in the gene encoding the 30S ribosomal binding factor RbfA.

Many other molecular tools have been applied to the overexpression of recombinant proteins. Qing and coworkers [155] constructed a series of cold-shock expression platforms (pCold I-IV vectors) regulated by the *cspA* promoter, the *cspA* 5′-UTR, and the *cspA* 3′ end transcription terminator site of *E. coli*. The expression platforms contain the *lac* operator sequence upstream of the *cspA* TSS to regulate the basal expression of the cloned genes at 37 °C. In addition, these vectors have a translation-enhancing element (TEE) and an optimized SD sequence (GAGG) intended to increase the expression by ~50% (average) in comparison with the unoptimized SD sequence (AAGG). In the Qing study, the pET14 expression vector carrying a T7 promoter was compared with the pCold-I expression vector carrying the optimized *cspA* promoter to express 38 proteins from *E. coli*, *Caenorhabditis elegans*, *Drosophila melanogaster*, and *Homo sapiens*. There was no substantial difference in recombinant protein expression and solubility for most proteins, regardless of the expression vector used. However, the expression and/or solubility of some proteins increased when the pCold-I expression vector was used. Remarkably high yields of the soluble proteins, i.e., the *E. coli* EnvZ ATP-binding domain (EnvZ-B) and the *Xenopus laevis* calmodulin (CaM), both aggregation-prone proteins, were reported with the use of the pCold expression vector. More recently, Liu et al. [156] found that during the expression of PACRG (Parkin co-regulated gene), the pCold system produced about 15–20 times more protein than the pET system. These results highlight the value of the pCold expression vector as a complementary alternative to the pET expression vector.

Moreover, the parallel use of affinity and solubility tags with recombinant DNA techniques may serve to modify proteins of interest and expedite their identification, production, or isolation from the host system. For example, Inouye and coworkers [157] reported that apoaequorin (AQ) from *Aequorea victoria* was not effectively expressed at 37°C as it forms protein aggregates in the cytoplasm of *E. coli* cells. Inouye and Sahara [158] expressed at 15°C the AQ fused to the solubility tag, named ZZ domain (IgG-binding domain of protein A), under the control of the *cspA* promoter. The fusion protein ZZ-AQ was mainly present in the soluble fraction (100% luminescence activity) of *E. coli* cells, whereas AQ without the ZZ domain (9.2% luminescence activity) was essentially produced as inclusion bodies. The authors suggested that the ZZ domain may function as a solubilization partner when the cold-induced expression is performed by *E. coli* cells.

Lin et al. [149] compared the expression of manganese peroxidases from *Ceriporiopsis subvermispora* (CsMnPs) in *E. coli* using the pET and pCold vectors. The system controlled by the T7 promoter produced 100% of the recombinant protein as inclusion bodies at 37 °C, but marginally soluble protein was obtained at 16 °C although with a low expression. In comparison, the system controlled by the *cspA* promoter produced high expression and 50% soluble protein at 15 °C. Protein solubility improved to ~ 100% by co-expressing the target gene with the folding accessory protein of the disulfide bond isomerase C (DsbC).

The commercial *cspA* promoter-TF system directs the expression of a trigger-factor (TF), a major ribosome-associated chaperone of *E. coli* involved in protein folding. The fusion-associated protein, TF, increases the solubility of the target protein, thus facilitating the co-translational folding of the expressed protein [55, 159, 160]. Hu et al. [55] contrasted the endoglucanase (EG) expression by the T7 expression system with that of the *cspA* expression system fused to a TF partner by cloning the *BpEG01790* from *Burkholderia pyrrocinia* B1213. Although *BpEG01790* was cloned into the conventional pET28a(+) vector under the control of the T7 promoter, no EG expression was detected with the *E. coli* BL21 (DE3) host system. Subsequently, *BpEG01790* was cloned in a pCold-TF vector directed by the *cspA* promoter and co-expressing a TF, which allowed successfully overexpressing an active BpEG01790 in *E. coli* BL21 cells (DE3). Moreover, the optimization of the culture medium improved the enzyme activity 12.5-fold. The application of the pCold system has also increased the solubility of other aggregation-prone proteins and enabled the successful expression of difficult-to-express proteins in *E. coli* [161–163]. Consequently, cold-induced promoters are valuable alternative genetic tools to the widely used T7 or *tac* promoters, to optimize the expression and solubility of certain proteins.

Another interesting possibility is the production of psychrophilic enzymes using cold-shock systems. The psychrophilic enzymes exhibit intrinsic instability at mesophilic growth temperatures but may be produced using engineered low-temperature expression systems. For example, the cold-shock induction of recombinant Arctic environmental genes was formerly reported by Bjerga and Williamson [164].

The cold shock response of *B. subtilis* has been extensively studied due to its remarkable adaptability and ability to survive at low temperatures in its natural habitat, the upper soil layer [8]. Le and Schumann [48] developed a cold-inducible expression platform for *B. subtilis*, based on the *des* promoter (*des* encodes a membrane lipid desaturase). Cold induction prevented the formation of protein aggregates and yielded much higher activity. Welsch and coworkers [8] optimized this expression platform by incorporating cold-inducible gene regulatory elements from *B. subtilis* and expressing two reporter genes encoding difficult-to-express proteins. The β-galactosidase encoding sequence, *TAE79A*, from the cold-adapted *Pseudoalteromonas haloplanktis*, when fused to the “downstream box” sequence of the *cspB,* resulted in considerably increased expression. This construct was fused with the *B. subtilis cspB* 5'-UTR sequence resulting in a further rise of β-galactosidase expression. Moreover, a further boost of expression and improved transcript stability were attained by incorporating, downstream the reporter gene, the transcription terminator of the *B. subtilis* cold-inducible *bkd* operon. Since the model protein *α*-glucosidase from *S. cerevisiae* can readily form inclusion bodies, it was used with the optimized platform in *B. subtilis* to validate the production of large amounts of soluble and functional α-glucosidase; the enzymatic activity attained was 1400 U L^−1^ at 20 °C, contrasting with 155 U L^−1^ at 37 °C. Suitably, this work shows the successful overproduction of poorly soluble proteins and enzymes, in the *B. subtilis* expression system regulated by the optimized *des* promoter, which is not feasible in established host expression systems such as *E. coli* [8].

The *hutU* and its upstream region of the Antarctic psychrophilic bacterium *Pseudomonas syringae* Lz4W have also been studied. It was found that *hutU* is inducible by a drop from 22 to 4 °C and that it operates more than one TSS. One initiation site was specific to cells grown at 4 °C but distinct from the common initiation sites identified at 4 °C and 22 °C. The typical promoter consensus sequences, containing the characteristic CAAAA sequence at the -10 position, were not found. However, the *hutU* mRNA was found to contain a long 5′-UTR, like those known in many cold-induced genes in mesophilic bacteria [9, 107]. Although Antarctic-adapted bacteria were discovered long ago, their genetic manipulation has so far been limited hitherto, accordingly little information is available on their transcriptional machinery. Two promoter consensus sequences (-35 box: TRGRTW and -10 box: TATRAY) of the psychrophilic bacterium *P. haloplanktis* TAC125 (*Ph*TAC125 strain) were identified by sequence comparison of 11 housekeeping promoters. This was facilitated by developing a shuttle genetic system to transform the *Ph*TAC125 strain. The identification and functional characterization of two upstream elements (UPs) from this bacterium were performed [165]. The endogenous plasmid pMtBL from the *Ph*TAC125 strain was molecularly characterized to recognize its efficient replication function. A pMtBL-derived cold expression platform was built, i.e., the first expression platform from a cold-adapted bacterium capable to produce thermolabile proteins at low temperatures [9, 166]. Colarusso et al. [167] used a pMtBL-deficient strain of *Ph*TAC125 (called KrPL) to obtain a KrPL *lacY*^*+*^ mutant strain, which was intended as an expression system for the recombinant production of difficult-to-express proteins (including eukaryotic proteins) at low temperatures (even at 0 °C) using the IPTG-inducible plasmid pP79. The engineered KrPL *lacY*^*+*^ strain produces a lactose permease and a truncated form of Lon protease, which enhances the internalization of IPTG and decreases the proteolytic events in the novel host system. The pP79 plasmid contains the *lacZ* promoter from *P. haloplanktis* TAE79 (*Ph*TAE79), a *Ph*TAE79 AraC family transcriptional regulator to confer stability to the target gene transcript, and the pMtBL-derived replication origin (*oriR*) for its maintenance in *Ph*TAC125. The expression of the recombinant β-galactosidase from *Ph*TAE79 using the expression system KrPL *lacY*^*+*^-pP79 showed higher protein yield in a soluble and active form at 15 and 0 °C.

Unlike cold-shock expression systems, psychrophilic expression systems show lower productivity of recombinant protein expression, which correlates with their longer doubling time (24 h to weeks). However, these expression systems may be regarded as further alternatives to produce recombinant proteins that are difficult-to-express in soluble and active form. However, it would be necessary to optimize the host systems of psychrophilic microorganisms and their genetic toolboxes.

Although successful low-temperature expression directed either by the *cspA* promoter from *E. coli* or the *des* promoter from *B. subtilis* has been previously performed and applied for the expression of proteins from bacteria to plants and humans, most likely, it would be imperative to develop other host expression systems to generate specific characteristics required on target proteins.

### Cold-responsive for protein expression in yeasts

Some studies have shown that low-temperature expression of recombinant proteins from diverse origins (prokaryotes to higher eukaryotes), in *P. pastoris*, can increase the yield, activity, stability, secretion, and solubility [168–174]. For example, heterologous expression of *Vitreoscilla* hemoglobin (VHb) improves cell growth and recombinant protein production in various hosts, including *P. pastoris*. In this yeast, the heterologous expression of VHb at 23 °C increased the final cell density and viability as compared with cells grown at 30 °C. In addition, the co-expression of VHb and recombinant β-galactosidase, at 23 °C, resulted in a higher rate of oxygen consumption and higher β-galactosidase levels, compared to cultures performed at 30 °C [173]. Likewise, the cells grown at 23 °C exhibited 2-fold higher VHb activity in comparison to those grown at 30 °C. Comparably, the secretion of the recombinant lipase Lip2 from the yeast *Candida rugose* was increased 32-fold when expressed in *P. pastoris* at low growth temperature combined with a selection antibiotic. The authors suggested that this strategy could be useful to increase yields for other lipases [175]. In addition, the soluble and biologically active herring antifreeze protein can be dramatically increased by its expression at low temperatures in *P. pastoris* [176]. The authors suggested that this result might be linked to an upgraded protein folding pathway and/or increased cell viability at low temperature. On the other hand, in *P. pastoris* our workgroup expressed ATP citrate lyase, a homotetrameric enzyme from *Phaffia rhodozyma* involved in the biosynthesis of lipids and carotenoids. However, the active form of the enzyme expressed was only detected when the recombinant yeast was grown at 25 °C and 300 rpm. The authors suggested that the production of the active recombinant enzyme may demand post-translational modifications such as phosphorylation and/or a suitable folding [168, 177]. Some other studies have reported successful expression at low temperatures in *S. cerevisiae*, *S. pombe, Kluyveromyces marxianus*, and *Y. lipolytica* [178–182].

Unlike bacteria, there are no commercial genetic platforms available in yeasts to direct the expression of target genes under cold regulation. However, the development of yeast vectors regulated by cold-shock promoters is beginning to gain research interest, mainly due to the striking results described above for *E. coli*. Bartolo-Aguilar et al. [120] constructed a functional cold-shock genetic vector for *P. pastoris* under the regulation of the *S. cerevisiae CCTα* promoter. This work proved that promoters induced by cold shock can be useful to design genetic platforms, to produce recombinant proteins in yeasts, that favor the establishment of white biotechnology strategies.

### Cold-responsive genetic promoters for protein expression in mammalian cells

Low temperatures are known to enhance the production of recombinant proteins in mammalian cell cultures, particularly those that are difficult to express at physiological temperature [133, 139]. Like in yeasts, there are still no reports of cold-inducible commercial genetic platforms for expressing recombinant proteins in mammalian cells. Nonetheless, some studies on the regulatory sequences of cold-inducible genes in mammals are available (Table 3). Thaisuchat and coworkers [183] disclosed a novel low temperature-sensitive promoter, named CHO S100a6, which yields 2- to 3-fold increase in basal transcript productivity in comparison with the promoter control SV40, after a drop from 37 to 33 °C. The CHO S100a6 promoter was detected after recognition of abundantly transcribed genes from CHO microarray expression data. The promoter sequence consisted of a TSS, TATA box, and several TFBs sited within the 1.5 kb upstream region of the ATG start signal. Moreover, CIRP is an evolutionarily conserved RNA-binding protein that is transcriptionally upregulated at low temperatures [23, 184]. Al-Fageeh and Smales [80] described the *CIRP* cold-shock promoter from mouse NIH-3T3 cells. Several genetic elements were identified within the 5'-UTR sequence of *CIRP* mRNA (a highly conserved mRNA within mammalian species), including TFBs. The core *CIRP* promoter (termed promoter 1 or P1) comprises the basal *CIRP* transcriptional regulatory elements within a 264 bp upstream region of the TSS. A second promoter was identified in the region -452 to -264 from the TSS (termed promoter 2 or P2). P2 was suggested to be an alternative promoter that can drive the transcription of the reporter gene independently of the core promoter. The authors showed that the putative promoters P1 and P2 do not function synergistically or additively. Luciferase reporter gene expression under P1, P2, or P1P2 regulation showed that the two promoters similarly respond to mild hypothermia; that is, luciferase activity increased 2-fold compared to 37 °C. The two single promoters both upregulate *CIRP* expression but produce alternative transcripts in response to mild hypothermia. Since this alternative promoter improves transgene expression using a reporter gene approach, the authors suggested its possible application to boost the recombinant gene expression at reduced temperatures in mammalian cells. Other CHO cold-inducible promoters and their regulatory control elements have been described and characterized after examining the utility of a cold shock-inducible promoter for low-temperature expression [145, 185, 186].

**Fig. 3:**
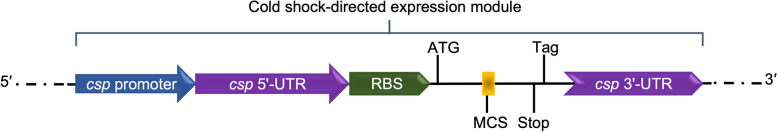
Schematic representation of an expression module for a cold-shock vector. Cold-shock proteins (CSPs) are found in all organisms. The promoters of upregulated *csp* genes, ribosome binding site (RBS), and transcription terminator are the primary genetic elements regulating cold shock-directed expression. The two former elements are sited on the upstream *csp* 5′-end, whereas the third element is sited downstream on the *csp* 3′-end. In addition to these genetic elements, the expression module must also contain the translation start codon (ATG), the multiple cloning sites (MCS), and the translation stop codon (Stop). Optionally, it can have some tags, such as the 6xHis tag, to facilitate the purification of recombinant proteins. *csp* 5′-UTR: 5′-untranslated region of *csp*, *csp* 3′-UTR: 3′-untranslated region of *csp*. Adapted from Bjerga and Williamson 2015

The cold-shock regulatory sequences harbor gene elements for transcription, so they could be useful for designing and constructing gene expression platforms to produce recombinant proteins. Fig. 3 shows a proposed expression module for the generation of cold-induced vectors.

## Perspectives

The increasing knowledge about microorganisms and mammalian cells has made possible the production of many products for the benefit of mankind. The use of DNA technology allowed recombinant proteins to enter the market, radically changing the pharmaceutical industry’s landscape [187]. Moreover, advances in biochemical analysis technology and recording this information in large biotechnological databases prompted the massive analysis of large amounts of data and enabled the identification of genetic characteristics with potential biotechnological application, including the development and optimization of genetic tools to produce recombinant proteins. The major regulatory sequences that promote transcription under cold conditions are sited in the upstream and downstream regions of native genes overexpressed at low temperature. The 5′-UTRs and 3′-UTRs sequences of transcripts have been regarded to regulate transcription stability and generate secondary structures that promote mRNA translation by allowing ribosome docking [8, 73, 74].

Some genes that are differentially expressed under cold conditions have been studied in *E. coli*, *S. cerevisiae*, and mammalian cells (*cspA*, *CCTα,* and *CIRP* respectively) [14, 23, 80, 119]. The *E. coli cspA* 5′-UTR has been reported to function as a thermosensor allowing rapid cellular adaptation to low temperatures through CspA overexpression. Comparably, the *E. coli cspA* 3′-UTR confers transcription stability [96, 188].

Cells confronting a cold shock decrease global transcription and translation, which temporarily leads to slower growth or even cell growth arrest while only a definite gene group becomes activated. The identification of native promoters of this group of cold-inducible genes makes feasible their application for recombinant protein production via the development of novel cold-inducible genetic platforms [80, 120, 145, 147, 155].

Cold response promoters have been scarcely studied in yeasts and mammalian cells, which limits their full development potential to produce recombinant proteins with these tools. Even so, the unique commercial genetic platform and its variants, available today to overexpress recombinant proteins under cold-shock conditions, have provided remarkable results in *E. coli*. That is, reduced inclusion bodies formation, the production of difficult-to-express proteins in a soluble and active form, and the expression of toxic proteins to cells [55, 155]. Initial research about cold shock-directed expression platforms for recombinant protein production suggests a promising future for protein biotechnology and the production of proteins that have not been readily expressed so far.

Moreover, the only available study with yeasts, performed in our laboratory, may well be regarded as a cornerstone for further development of expression platforms and bioprocesses that use cold-sensitive promoters. The cold-responsive *CCTα* promoter of *S. cerevisiae* was functional in *P. pastoris* to direct the expression of endoglucanase that degrades the Pichia cell wall [120]. Accordingly, yeast cells having this expression platform self-autolyze in response to a cold shock, which may ease the recovery and reduce the costs of producing recombinant proteins. Then, the development of commercial platforms directed by cold-sensitive promoters to express recombinant proteins in yeasts and mammalian cells appears promising. These eukaryotic systems are mostly used to express relatively large recombinant proteins (> 50 kDa) and to incorporate certain required post-translational modifications. On the other hand, future studies to identify a wider variety of cold-inducible promoters from psychrophilic organisms are warranted [117, 189]. In our opinion, this will greatly help to develop cold-inducible expression platforms and to understand the molecular mechanisms involved in adaptation to cold.

The production of recombinant proteins lacking solubility tags appears compulsory, as fusion tags may elicit a host immune response, in vivo treatments [163]. The use of expression vectors directed by cold-induced promoters seems advantageous, since they have already been shown to improve the solubility and activity of some target proteins. Another promising use of cold-responsive promoters involves the production of biological products via metabolic engineering, that is by remodeling and controlling metabolic pathways by means of cold-response switches [147].

Ultimately, producing recombinant proteins in different host expression systems regulated by cold shock will significantly contribute to the establishment of bioprocesses based on the principles of white biotechnology.

## Conclusions

Our review concludes that cold-induced promoters are suitable and valuable tools to produce recombinant proteins, regardless of their use with bacteria, yeasts, or mammalian cells. Proteins of pharmaceutical interest would be attractive to produce using cold shock expression platforms, compared to their production with conventional platforms, particularly those prone to aggregation or those inherently labile. Such systems may also be useful for expressing toxic (or harmful) proteins to the host, psychrophilic proteins, or thermolabile proteins. The use of eukaryotic cold shock promoters for sure will contribute to the application of white bioprocess technology for recombinant protein production. Several cold-induced promoters appear promising to generate novel expression vectors in combination with existing synthetic biology toolboxes. Furthermore, protein expression at low temperatures but without chemical inducers, in the end, seems advantageous for production at large-scale biotechnological processes. Remarkable advances in protein biotechnology through expression vectors directed by cold-shock promoters are limited to the *E. coli* expression system. Future use of cold-sensitive promoters for recombinant protein production in yeasts and mammalian cells will allow the expression of difficult-to-express proteins.

## Abbreviations

AOX1: Alcohol oxidase 1
AQ: Apoaequorin
*araBAD*: Arabinose operon
ATG: Translation start codon
CaM: Calmodulin
CAP: Catabolite activator protein
CCT: Chaperonin containing the T-complex polypeptide-1 [TCP1]
cDNA: Complementary DNA
CHO: Chinese hamster ovary
CIRP: Cold-inducible RNA-binding protein
CMV: Cytomegalovirus
CSD: Cold-shock domain
CSP: Cold-shock protein
DNA: Deoxyribonucleic acid
DsbC: Disulfide bond isomerase C
EnvZ-B: EnvZ ATP-binding domain
FDA: Food and Drug Administration
GAL1: Galactokinase
GAL4-E1b: The adenoviral E1B minimal core promoter fused to DNA-binding sites for the yeast GAL4 DNA-binding protein
GAP: Glyceraldehyde-3-phosphate dehydrogenase
GRAS: Generally recognized as safe
HEK: Human embryonic kidney
IPTG: β-D-1-thiogalactopyranoside
IRES: Internal ribosomal entry sites
LAC4: β-galactosidase
MCS: Multiple cloning sites
mRNA: Messenger RNA
ORF: Open reading frame
PACRG: Parkin co-regulated gene
qRT-PCR: Quantitative real-time polymerase chain reaction
RBM3: RNA-binding motif protein 3
RBS: Ribosome binding site
RNA: Ribonucleic acid
rRNA: Ribosomal RNA
SD: Shine-Dalgarno
sRNA: Small RNA
T5/*lac*: *lac* operator sequence just downstream of the T5 promoter
T7: T7 RNA polymerase
*tac*: The *tac* promoter is a synthetic DNA promoter, produced from the combination of promoters from the *trp* and *lac* operons
TEEs: Transcription enhancer elements
TF: Trigger-factor
TFBs: Transcription factor binding sites
TFIID: Transcription factor IID
*trc*/*lac*: *trc* promoter is a hybrid between the *trp* (tryptophan) and *lac*UV5 promoters containing de *lac* operator
TSS: Transcription start site
uAUGs: Upstream AUGs
uORFs: Upstream open reading frames
UP: Upstream element
UTR: Untranslated region
VHb: Vitreoscilla hemoglobin
ZZ: IgG-binding domain of protein A
